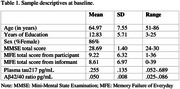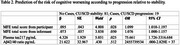# Predicting cognitive worsening using subjective reports of dyads and biological biomarkers in pre‐clinical stages: Preliminary results from the CompAS cohort study

**DOI:** 10.1002/alz70856_104822

**Published:** 2026-01-07

**Authors:** Lucía Pérez‐Blanco, Ana I. Rodríguez‐Pérez, Alba Felpete, Ana Nieto‐Vieites, Fátima Fernández‐Feijoo, Cristina Lojo‐Seoane, Onésimo Juncos‐Rabadán, David Facal, Arturo X. Pereiro Rozas

**Affiliations:** ^1^ Applied Cognitive Neuroscience and Psychogerontology group, Health Research Institute of Santiago de Compostela (IDIS), Santiago de Compostela, Spain; ^2^ Departamento de Psicoloxía Evolutiva e da Educación, Universidade de Santiago de Compostela, Santiago de Compostela, Galicia, Spain; ^3^ Instituto de Psicoloxía (IPsiUS), Universidade de Santiago de Compostela, Santiago de Compostela, Galicia, Spain; ^4^ Instituto de Investigación Sanitaria de Santiago de Compostela (IDIS), Santiago de Compostela, Galicia, Spain; ^5^ Centro Singular de Investigación en Medicina Molecular e Enfermidades Cronicas (CIMUS), Universidade de Santiago de Compostela, Santiago de Compostela, Galicia, Spain; ^6^ Centro de investigación Biomédica en Red sobre Enfermedades Neurodegenrativas (CIBERNED), Santiago de Compostela, Gallicia, Spain; ^7^ Departamento de Psicoloxía Evolutiva e da Educación, Universidade de Santiago de Compostela, Santiago de Compostela, Spain; ^8^ Instituto de Psicoloxía (IPsiUS), Universidade de Santiago de Compostela, Santiago de Compostela, Spain

## Abstract

**Background:**

The utility of cognitive complaints and biological biomarkers as a factor risk of dementia among cognitively unimpaired individuals is not yet clear (Nosheny et al., 2022). Our aim was to study the joint contribution of subjective reports and some plasma biomarkers to predict cognitive worsening from preclinical stages.

**Method:**

One‐hundred older adults identified as CU and SCD from the CompAS study, and their corresponding informants, were followed with an average time of 59.86 months (range=47‐77; SD=7.36). After an extensive neuropsychological assessment, they were diagnosed by applying consensus criteria. The Memory Failure of Everyday (MFE), a 28‐item questionnaire (Sunderland et al., 1984), was administered at baseline to participants and informants for assessing memory complaints and plasma biomarkers *p*‐tau217, Aβ42 and Aβ40 were obtained from participants (see Table 1). Logistic regression analysis was used to predict stability and worsening towards MCI or dementia at last follow‐up, using total MFE scores (self‐report and informant) and plasma tau‐217 and Aβ42/Aβ40 ratio as predictors.

**Results:**

Regression model showed significant predictive associations between worsening towards MCI or dementia and higher participant MFE total‐score and Plasma *p*‐tau levels, (see Table 2). The informant MFE total‐score was close to achieve significance (*p* = .050) and Aβ42/40 ratio failed in predicting progression. The model fitting parameters showed acceptable fits (*χ^2^* = 14.267, *p* = .014; *Nalgelkerke R^2^* = .203; Hosmer & Lemeshow test= 5.713; *p* = .679). Classification values were: Sensibility=21.1, Specificity=98.8, and Overall percentage=84.0.

**Conclusions:**

Results showed that higher MFE score from participants and levels of plasma *p*‐tau217 were associated to increased risk of cognitive worsening in pre‐clinical stages. These results should be considered with caution given that the model classified better the stable than the worsening participants.